# Effect of Commonly Used Pediatric Antibiotics on Gut Microbial Diversity in Preschool Children in Burkina Faso: A Randomized Clinical Trial

**DOI:** 10.1093/ofid/ofy289

**Published:** 2018-11-02

**Authors:** Catherine E Oldenburg, Ali Sié, Boubacar Coulibaly, Lucienne Ouermi, Clarisse Dah, Charlemagne Tapsoba, Till Bärnighausen, Kathryn J Ray, Lina Zhong, Susie Cummings, Elodie Lebas, Thomas M Lietman, Jeremy D Keenan, Thuy Doan

**Affiliations:** 1 Francis I. Proctor Foundation; 2 Department of Ophthalmology, University of California, San Francisco, California; 3 Department of Epidemiology and Biostatistics, University of California, San Francisco, California; 4 Centre de Recherche en Santé de Nouna, Nouna, Burkina Faso; 5 Heidelberg Institute of Public Health, Heidelberg, Germany; 6 Africa Health Research Institute, Somkhele, South Africa; 7 Department of Global Health and Population, Harvard School of Public Health, Boston, Massachusetts

**Keywords:** antibiotics, microbiome, randomized controlled trial, sub-Saharan Africa

## Abstract

**Background:**

Exposure to antibiotics may result in alterations to the composition of intestinal microbiota. However, few trials have been conducted, and observational studies are subject to confounding by indication. We conducted a randomized controlled trial to determine the effect of 3 commonly used pediatric antibiotics on the intestinal microbiome in healthy preschool children.

**Methods:**

Children aged 6–59 months were randomized (1:1:1:1) to a 5-day course of 1 of 3 antibiotics, including amoxicillin (25 mg/kg/d twice-daily doses), azithromycin (10 mg/kg dose on day 1 and then 5 mg/kg once daily for 4 days), cotrimoxazole (240 mg once daily), or placebo. Rectal swabs were obtained at baseline and 5 days after the last dose and were processed using 16S rRNA gene sequencing. The prespecified primary outcome was inverse Simpson’s α-diversity index.

**Results:**

Post-treatment Simpson’s diversity was significantly different across the 4 arms (*P* = .003). The mean Simpson’s α-diversity among azithromycin-treated children was significantly lower than in placebo-treated children (6.6; 95% confidence interval [CI], 5.5–7.8; vs 9.8; 95% CI, 8.7–10.9; *P* = .0001). Diversity in children treated with amoxicillin (8.3; 95% CI, 7.0–9.6; *P* = .09) or cotrimoxazole (8.3; 95% CI, 8.2–9.7; *P* = .08) was not significantly different than placebo.

**Conclusions:**

Azithromycin affects the composition of the pediatric intestinal microbiome. The effect of amoxicillin and cotrimoxazole on microbiome composition was less clear.

**Clinical Trials Registration:**

clinicaltrials.gov NCT03187834.

Exposure to antibiotics in childhood is thought to alter the intestinal microbiome [1–3]. Observational studies in high-income settings have suggested that there are alterations in the intestinal microbiome in children receiving antibiotics relative to those who have not received antibiotics [3–5]. Antibiotics disrupt the composition of microbiota as they have activity against both commensal and pathogenic bacteria [2]. Dysbiosis of the intestinal microbiome has been linked to multiple adverse health outcomes, including undernutrition and obesity, asthma, diabetes, and some forms of cancer [6–14].

A recent randomized controlled trial evaluated the effect of a single dose of azithromycin, as is used for mass distribution in trachoma control programs [15–17], on the composition of the intestinal microbiome among preschool children in Niger [1]. This study found a significant decrease in the diversity of the intestinal microbiome in a population of relatively antibiotic-naïve children. In many regions of sub-Saharan Africa, macrolides are used less frequently than penicillins or sulfonamides for the treatment of common childhood illnesses [18–20]. Here, we assess the effect of a short course of 3 commonly used pediatric antibiotics on the intestinal microbiome. We hypothesized that all 3 antibiotics would lead to decreased diversity in the intestinal microbiome compared with placebo.

## METHODS

### Study Setting

This study took place in 2 rural communities of the Health and Demographic Surveillance Site (HDSS) in Nouna District, Burkina Faso [21, 22]. A triannual census is performed in the HDSS by the Centre de Recherche en Santé de Nouna (CSRN). Inhabitants of the study communities are primarily subsistence farmers and cattle keepers. The study occurred in July 2017, just before the rainy season [23].

### Participants and Procedures

The overall objective of the study was to investigate the direct and indirect effects of antibiotic usage on the intestinal microbiome of preschool-aged children. We recruited households in the 2 study communities with 2 or 3 children between the ages of 6 and 59 months based on the most recent HDSS census. In households with 3 children, all children received a study drug but only 2 randomly selected children were monitored as part of the study. Children were eligible for participation in the study if they were between 6 and 59 months of age and with parental consent. We did not exclude children on the basis of preexisting morbidity or recent antibiotic use. Children were assessed before randomization (baseline) and then again 5 days after the last antibiotic dose (post-treatment).

### Randomization

We employed a 2-stage randomization procedure. First, each household was randomized in a 1:1:1:1 fashion to 1 of the 4 study arms: 1) amoxicillin, 2) azithromycin, 3) co-trimoxazole, or 4) placebo. Second, each child in the household was randomly assigned to either treatment or placebo. In households with 2 children, 1 was randomized to treatment and the other to placebo. In households with 3 children, 2 were randomized to treatment and the other to placebo. Note that in the placebo households, children assigned to treatment and placebo received the same drug (ie, placebo). The present report is concerned with the direct effects of antibiotics and therefore only includes the 1 child per household randomized to treatment. The randomization sequence was generated by TCP in R, version 3.3.1 (The R Foundation for Statistical Computing), using a masked seed value [24]. The randomization sequence was implemented in the field by preloading syringes with the child’s randomized treatment that were labeled with each child’s name (described below).

### Interventions

All study medications were prepared as pediatric oral suspension. Study medications were procured at local pharmacies in the study area, with the exception of azithromycin, which was procured in Ouagadougou. Weight measurements were collected during the baseline visit as part of anthropometry assessments. These measurements were used to calculate the appropriate dose of study medication for each child. There was no standard dosing for study medication as children were not being treated for an established infection. We therefore used the lower end of approved dosing regimens for each antibiotic to reduce the risk of adverse events. All antibiotics were administered for 5 days. We used the lower end of approved dosing for amoxicillin for children under 12 years of age (25 mg/kg/d in twice-daily doses). Azithromycin dosing was based on the lower end of the range for standard pediatric dosing for mild to moderate infection (a single 10-mg/kg dose on day 1 and then 5 mg/kg once daily for 4 days). Cotrimoxazole dosing was based on prophylactic dosing for children living with HIV (240 mg once daily) [25–28]. Placebo was prepared by study staff as a mixture of powdered milk, sugar, and bottled water. All study medications were prepared daily in orange opaque syringes and labeled uniquely for each child. Treatment was administered from a central point in each study community. A community mobilizer visited the homes of the children participating in the study and instructed the caregivers to bring the children to the central point for examination and treatment visits. Treatment teams recorded whether each child received the study treatment and reasons for a missed treatment.

### Masking

Treatment teams were not told the identity of the study medication, and the orange tinting of the syringes helped to conceal the identity of the study drug. However, due to differences in taste and appearance, the treatment team was not masked. In contrast, examination teams were masked to treatment assignment, and laboratory personnel were masked to treatment assignment and time point.

### Baseline Questionnaire

At baseline, the caregiver of each child completed a short questionnaire related to the child and the child’s household of residence. Health status questions included if the child had recently visited a health facility and, if so, if the child had been treated with antibiotics after the visit. Caregivers were asked if the child was currently breastfeeding.

### Outcome Assessment

Rectal samples were collected in the field at baseline and 5 days after the last antibiotic treatment. Examiners inserted a swab 1–3 cm into the anus and rotated 360 degrees. Examiners changed gloves between each participant. Swabs were immediately placed in a Stool Nucleic Acid Collection and Transport Tube containing Norgen Stool Preservative (Norgen, Ontario, Canada). The transport media preserves DNA and RNA in the sample and prevents growth of organisms. Samples were placed at ambient temperature in the field, and then stored at the CRSN laboratory at –80°C until they were shipped to the United States. Samples were shipped on ice and then stored at –80°C until processing. Samples were de-identified in the field and then placed in a random order for library preparation and sequencing. DNA was extracted from the fecal samples using the Norgen stool DNA isolation kit (Norgen, Ontario, Canada), per the manufacturer’s instructions. Concentration of DNA was quantified using the Qubit dsDNA HS Assay Kit (ThermoFisher Scientific, Waltham, MA) and adjusted to 15 ng/uL. The gut bacterial community was assessed by deep sequencing the V3-V4 hypervariable regions of the 16S rDNA gene. Library preparation was performed by SeqMatic (Fremont, CA) per the Illumina16S metagenomic sequencing library preparation protocol. Demultiplexed raw sequences were processed in QIMME, version 1.9, which utilizes the Ribosomal Database Project Classifier and the full GreenGenes 13_8 reference database to assign taxonomy to each sequencing read.

### Sample Size Determination

The sample size calculation was based on the primary outcome, Simpson’s α-diversity. A sample size of 30 children per arm was estimated to provide at least 80% power to detect a 1.5-unit difference in Simpson’s α-diversity based on a previous study in Niger [1].

### Statistical Methods

The primary outcome of the study was prespecified as α-diversity (inverse Simpson’s) at the genus level, expressed in effective number. Shannon’s α-diversity was calculated as a secondary outcome [29]. The primary prespecified analysis assessed mean post-treatment diversities, compared across all arms with an analysis of variance and pairwise comparisons performed with a *t* test. As a sensitivity analysis, the post-treatment diversities were compared between arms in a linear regression model adjusted for baseline diversity. As an additional sensitivity analysis, post-treatment diversities were compared between arms with a term for both the child’s age and the baseline diversity measurement in a linear regression model. We used permutational multivariate analysis of variance (PERMANOVA) to assess differences in microbial composition between arms using Manhattan and Euclidean distances. A principal coordinates analysis (PCoA) was used to visually depict the centroids of the groups. All *P* values were calculated using a Monte Carlo permutation test with 10 000 replications, and *P* values <.05 were considered statistically significant for all tests. All analyses were conducted in R, version 3.4.3 (The R Foundation for Statistical Computing). Diversity measures and Manhattan and Euclidean distances were calculated in the R package “vegan.”

### Ethical Considerations

The study was reviewed and approved by the Comité Institutionnel d’Ethique at the Centre de Recherche en Santé de Nouna in Nouna, Burkina Faso, and the Institutional Review Board at the University of California in San Francisco. Written informed consent was obtained from the caregiver of each participant. The trial was registered at clinicaltrials.gov (NCT03187834).

## RESULTS

**Figure 1.**
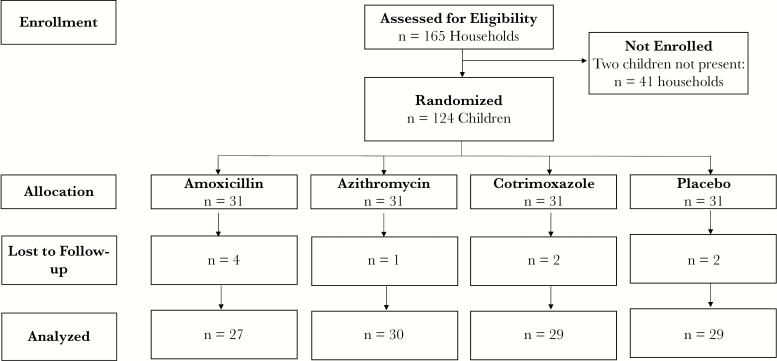
Consolidated Standards for Reporting of Trials study flow diagram.

In July 2017, 248 children in 124 households were enrolled and randomized to 1 of the 3 antibiotic regimens or placebo (Figure 1). Of these, 124 children were randomized within their household to receive treatment and are included in this analysis. Of these children, 120 (96.8%) had a rectal swab collected 5 days after their last antibiotic dose. The median age (interquartile range) was 36 (21–51) months, and 54.0% of the children were female (Table 1). One-quarter (27.4%) were currently breastfeeding, and 7.3% had received an antibiotic from a health facility in the last month. Baseline characteristics were well balanced across the 4 study arms. Adherence to study medication and adverse events have been previously reported [30]. More than 90% of children received their allocated study medication at most time points. Adverse events were uncommon, and no diarrhea was noted in any of the antibiotic arms. 16S rRNA gene analysis of the fecal samples identified 429 unique genera. At both baseline and post-treatment, the most common genus was *Prevotella* spp. (Supplementary Figure 1).

**Table 1.** Baseline Characteristics by Study Arm

	Amoxicillin (n = 31)	Azithromycin (n = 31)	Cotrimoxazole (n = 31)	Placebo (n = 31)
Age, median (IQR), mo	32 (23–48)	29 (21–51)	37 (23–48)	38 (23–51)
Female sex, No. (%)	15 (48.4)	18 (58.1)	20 (64.5)	14 (45.2)
Recently visited health facility, No. (%)	6 (19.4)	4 (12.9)	6 (19.4)	5 (16.1)
Recent antibiotic use, No. (%)	2 (6.5)	2 (6.5)	3 (9.7)	2 (6.5)
Breastfeeding, No. (%)	6 (19.4)	11 (35.5)	9 (29.0)	8 (25.8)

Abbreviation: IQR, interquartile range.

**Figure 2.**
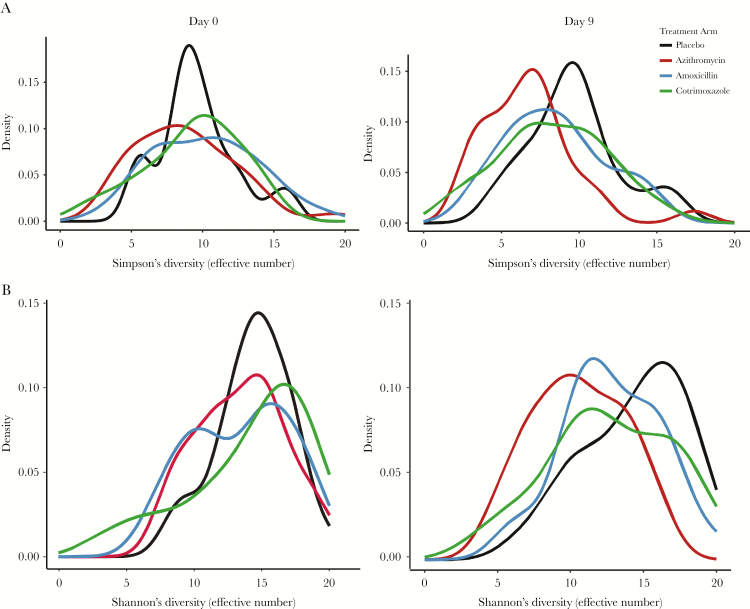
Distributions of Simpson’s (A) and Shannon’s (B) alpha diversity indices at baseline and 5 days after the final study treatment for children treated with placebo (black lines), azithromycin (red lines), amoxicillin (blue lines), and cotrimoxazole (green lines).

**Figure 3.**
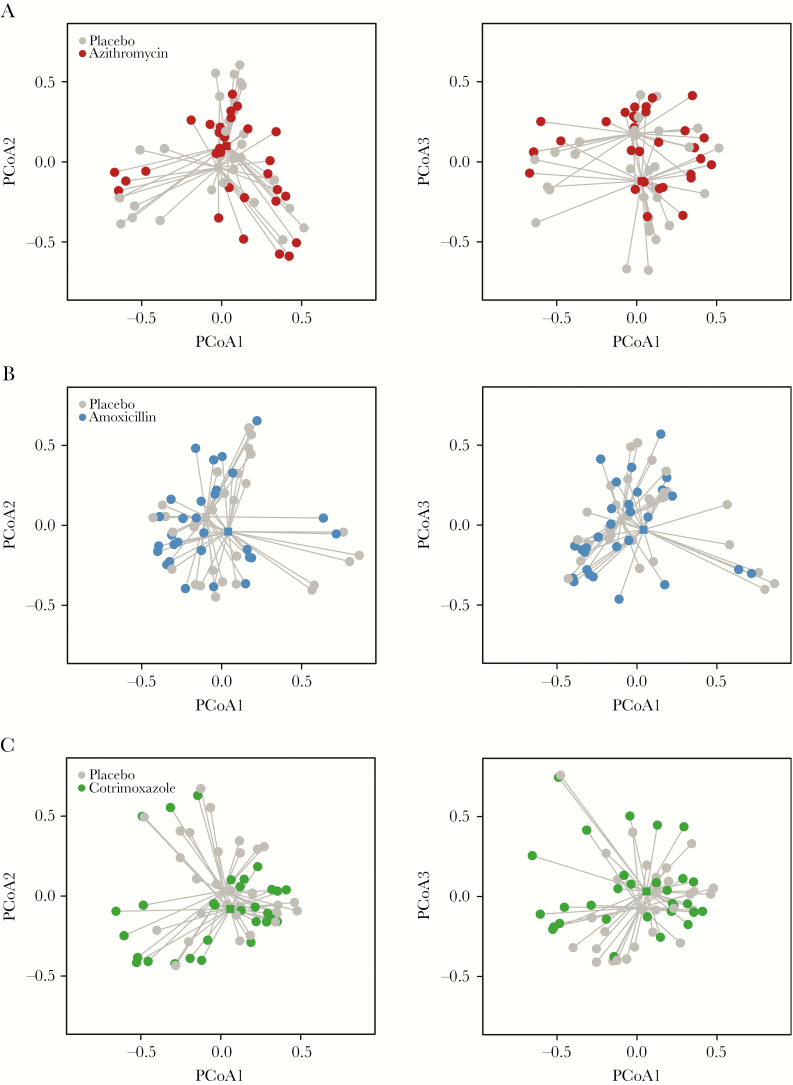
Principal coordinates analysis (PCoA) plots comparing the Euclidean distance between placebo and azithromycin (A), placebo and amoxicillin (B), and placebo and cotrimoxazole (C). Centroids are depicted with square points.

There was no difference in Simpson’s or Shannon’s α-diversity across the 4 study arms at baseline (*P* = .55 and *P* = .59, respectively) (Table 2). Five days after the last antibiotic dose, there was an overall difference in α-diversity across all arms, as measured by Simpson’s and Shannon’s diversities (*P* = .003 and *P* = .0001, respectively) (Table 2). Results were robust to adjustment for the child’s age (*P* = .005 for Simpson’s and *P* = .002 for Shannon’s diversity). The post-treatment mean Simpson’s α-diversity effective number was 6.6 (95% confidence interval [CI], 5.5–7.8) in the azithromycin arm, 8.3 (95% CI, 7.0–9.6) in the amoxicillin arm, 8.3 (95% CI, 6.9–9.7) in the cotrimoxazole arm, and 9.8 (95% CI, 8.7–10.9) in the placebo arm (Figure 2; Table 2). Simpson’s diversity was significantly reduced in the azithromycin arm compared with placebo (*P* = .0001 by *t* test, *P* = .0001 by linear regression). Simpson’s diversity index was nonsignificantly lower in children receiving amoxicillin (*P* = .09 by *t* test, *P* = .03 by linear regression) and co-trimoxazole (*P* = .08 by *t* test, *P* = .048 by linear regression) compared with placebo (Table 2). Differences in Shannon’s diversity index were similar to the primary outcome (azithromycin vs placebo: *P* = .0002; amoxicillin vs placebo: *P* = .09; cotrimoxazole vs placebo: *P* = .048; all *t* test) (Figure 2). Similarly, PERMANOVA analysis found a significant difference in L1 norm (equivalent to Shannon’s diversity, *P* = .01) and L2 norm (equivalent to Simpson’s diversity, *P* = .0001) across the study arms (Figure 3).

**Table 2.** Simpson’s and Shannon’s Alpha Diversity at Baseline and Post-treatment

	Amoxicillin		Azithromycin		Cotrimoxazole		Placebo	
	Baseline (n = 31)	Post-treatment (n = 27)	Baseline (n = 31)	Post-treatment (n = 30)	Baseline (n = 31)	Post-treatment (n = 29)	Baseline (n = 31)	Post-treatment (n = 29)
Simpson (inverse) effective number, mean (95% CI)	10.2 (8.8–11.5)	8.3 (7.0–9.6)	8.8 (7.5–10.1)	6.6 (5.5–7.8)	9.7 (8.2–11.2)	8.3 (6.9–9.7)	9.6 (8.6–10.7)	9.8 (8.7–10.9)
Shannon (exponential) effective number, mean (95% CI)	16.6 (14.5–18.7)	13.9 (12.1–15.8)	14.6 (13.0–16.2)	11.0 (9.3–12.7)	15.6 (13.4–17.8)	13.5 (11.6–15.4)	15.4 (14.1–16.7)	16.0 (14.3–17.8)

Abbreviation: CI, confidence interval.

## DISCUSSION

A course of azithromycin significantly reduced intestinal bacterial diversity and composition in children in Burkina Faso, consistent with a previous study in Niger [1]. In the previous study, children received a single dose of azithromycin or placebo, with the same dosing used by trachoma control programs and a recent trial investigating the effect of azithromycin on child survival [15, 16, 31, 32]. Here, we used a 5-day course with dosing equivalent to a Z-pack, a common course for treating mild infections in children such as otitis media. Taken together, the results of these studies suggest a definitive decrease in intestinal bacterial diversity following an azithromycin course in children.

The effect of amoxicillin or co-trimoxazole on intestinal bacterial diversity was less clear. Although there was a decrease in diversity between baseline and post-treatment for both antibiotics, the prespecified primary analysis was not statistically significant for either antibiotic. A sensitivity analysis including the baseline measurement for diversity yielded borderline significant results for both antibiotics, suggesting that there may be an effect of both antibiotics on the microbiome but that the present study may have been underpowered to detect smaller differences. A previous observational study among Finnish children showed a decrease in diversity with macrolides but not penicillins and suggested that macrolides had a greater effect on dysbiosis than pencillins [4]. In the current study, because antibiotics were not being given for established infection, amoxicillin dosing was chosen to be the lower end of the approved range to minimize side effects. Cotrimoxazole was dosed following World Health Organization guidelines for cotrimoxazole prophylaxis for children living with HIV [25, 26, 28]. Higher doses of cotrimoxazole or amoxicillin may yield different effects on the microbiome. Further, the condition in which the medications were stored may have altered their efficacy. Both cotrimoxazole and amoxicillin were purchased in Nouna, a rural area in Burkina Faso, where temperature monitoring of the medications was not observed. Therefore, higher doses of quality amoxicillin or cotrimoxazole may lead to greater changes in the composition of the intestinal microbiota. However, the use of locally sourced antibiotics simulates the conditions under which children in similar settings are treated, and thus these results are likely representative of intestinal microbiome changes following the use of similar antibiotics in west Africa.

Previous studies in high-income settings have indicated that dysbiosis of the intestinal microbiome may be associated with morbidity in children, including asthma [9], food allergy [33], and obesity [34]. In low-income settings, differences in intestinal diversity have been noted in children with different forms of severe acute malnutrition [12] and in twin pairs discordant for kwashiorkor [8]. Antibiotics have been shown to lead to weight gain in children in randomized controlled trials [30, 35]. Alteration to the intestinal microbiome induced by antibiotic use may affect nutrient absorption or energy metabolism that can affect weight gain in children [36, 37], or weight gain after antibiotic use may be mediated by reduction in the burden of enteropathogens. However, the clinical implications of a single course of antibiotics remain unclear.

Several limitations should be noted. The duration of follow-up in the present study was short, and thus evaluated short-term changes in the intestinal microbiome following a course of antibiotics. Some studies have indicated that there may be longer-term changes in the microbiome following antibiotic use in children [4]. It is possible that the changes observed in the present study are transient, or they may persist for several months. Recent antibiotic use was uncommon in the study population, but contamination of treatment assignment could have occurred if children used antibiotics other than the study medication during the course of the study. This study was conducted in a rural area of Burkina Faso that is characterized by high infection burden and high mortality. The results of this study may not be generalizable to children living in different settings, as the microbiome in children differs substantially geographically [38]. However, the results of this study support previous observational and randomized studies that have indicated effects on intestinal microbial diversity following antibiotic use [1, 4, 5, 39].

In this randomized controlled trial of 3 commonly used antibiotics, we demonstrated that a short course of azithromycin led to a significant decrease in bacterial diversity of the intestinal microbiome in preschool children. Amoxicillin and cotrimoxazole dosing consistent with that used for prophylaxis in children living with HIV did not lead to a significant difference in bacterial diversity. Although the clinical implications of a single course of antibiotics are unclear, the results of this study indicate that the intestinal microbiome in young children is sensitive to antibiotics.

## Supplementary Data

Supplementary materials are available at *Open Forum Infectious Diseases* online. Consisting of data provided by the authors to benefit the reader, the posted materials are not copyedited and are the sole responsibility of the authors, so questions or comments should be addressed to the corresponding author.

Supplementary Table 1
