# Supplementary figures and images for: Effect of Commonly Used Pediatric Antibiotics on Gut Microbial Diversity in Preschool Children in Burkina Faso: A Randomized Clinical Trial
Source: Open Forum Infect Dis. 2018 Nov 2;5(11):ofy289. doi: 10.1093/ofid/ofy289 (PMC6262116; doi:10.1093/ofid/ofy289)

## Baseline Visit

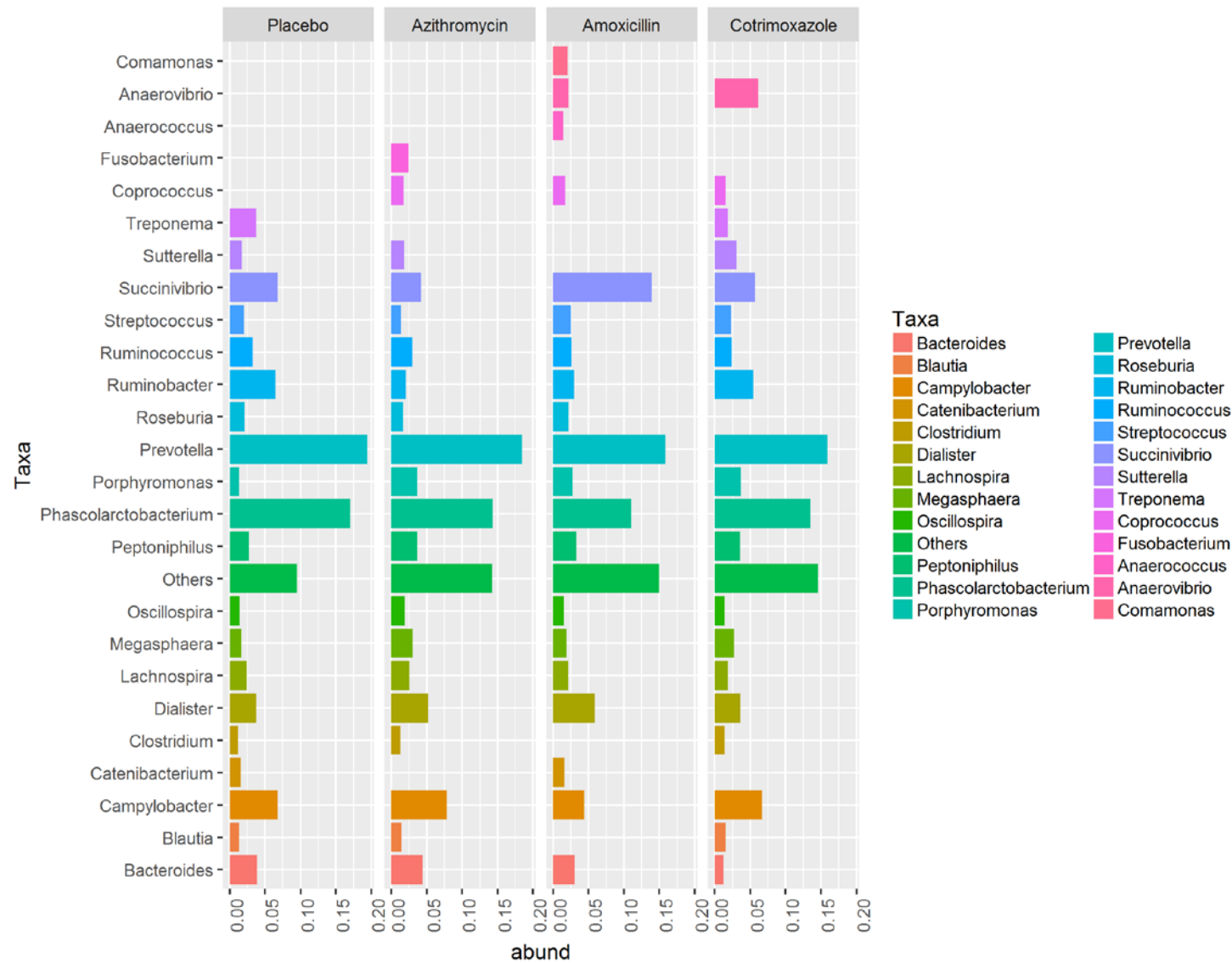

## 6-Day Visit

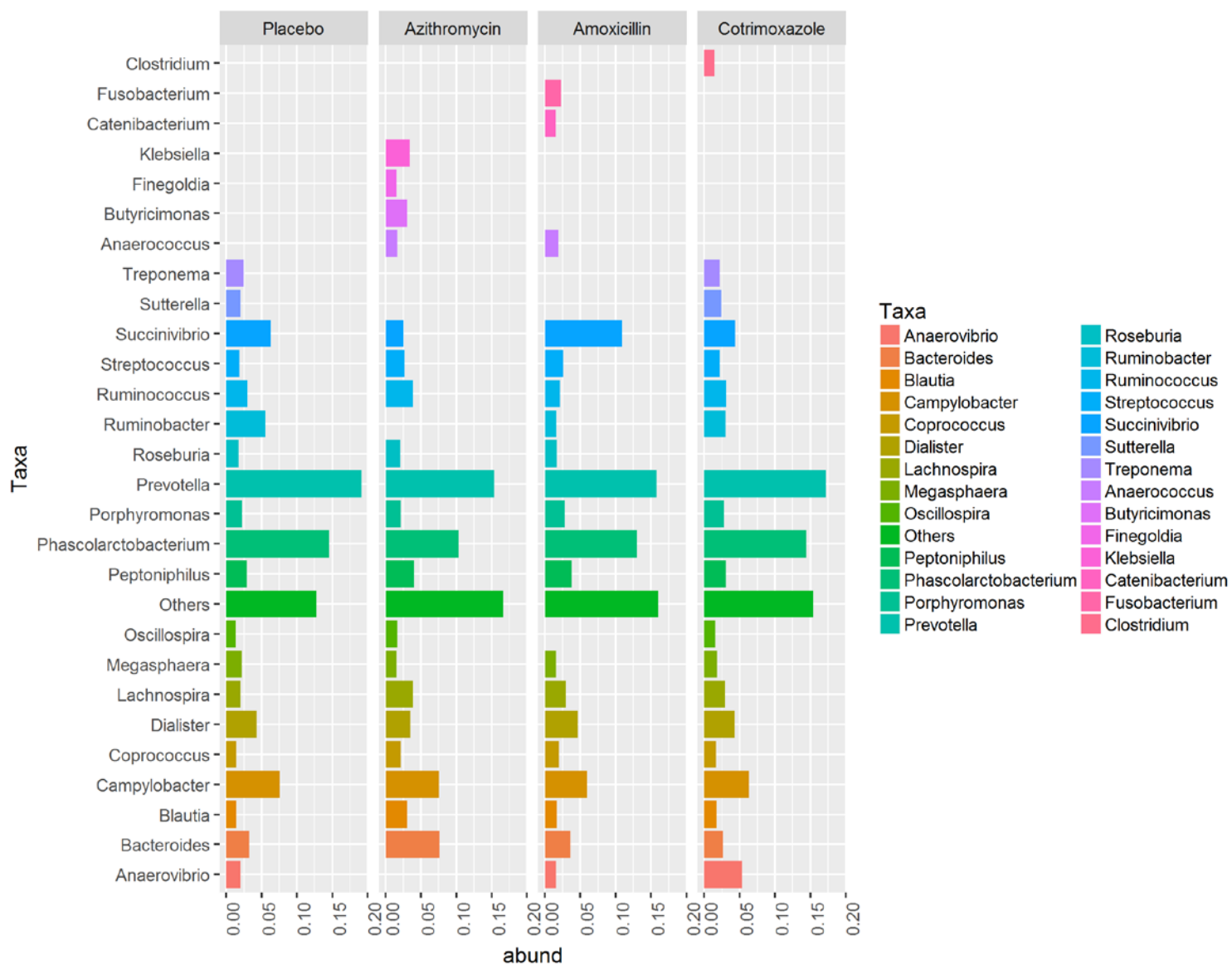

Supplement: Supplementary Table 1 [file ofy289_suppl_supplementary_table_1.pdf]
